# Diagnostics of rare disorders: whole-exome sequencing deciphering locus heterogeneity in telomere biology disorders

**DOI:** 10.1186/s13023-018-0864-9

**Published:** 2018-08-17

**Authors:** Luca Trotta, Anna Norberg, Mervi Taskinen, Vivien Béziat, Sofie Degerman, Ulla Wartiovaara-Kautto, Hannamari Välimaa, Kirsi Jahnukainen, Jean-Laurent Casanova, Mikko Seppänen, Janna Saarela, Minna Koskenvuo, Timi Martelius

**Affiliations:** 1 Institute for Molecular Medicine Finland FIMM, HiLIFE, University of Helsinki, P.O.BOX 281, FI-0029 Helsinki, Finland; 2 Department of Medical Biosciences, Medical and Clinical Genetics, Umeå University, Building 6M, SE-901 87 Umeå, Sweden; 3 Division of Hematology-Oncology and Stem Cell Transplantation, Children’s Hospital, University of Helsinki and Helsinki University Hospital, Haartmaninkatu 4, PL 372, 00029 HUS Helsinki, Finland; 4 Laboratory of Human Genetics of Infectious Diseases, Necker Branch, INSERM U1163, Necker Hospital for Sick Children, Paris, France; 5 Paris Descartes University, Imagine Institute, 24 boulevard du Montparnasse, 75015 Paris, EU France; 6 Department of Medical Biosciences, Pathology, Umeå University, NUS, Dept of Medical Biosciences M21, 901 85 Umeå, Sweden; 7 Department of Haematology, Helsinki University Hospital Comprehensive Cancer Center and University of Helsinki, Helsinki, Finland; 8 Faculty of Medicine Department of Virology and Department of Oral and Maxillofacial Surgery, University of Helsinki and Helsinki University Hospital, POB 21, 00014 Helsinki, Finland; 9 Department of Women’s and Children’s Health, Karolinska Institute and University Hospital, Stockholm, Sweden; 10 St. Giles Laboratory of Human Genetics of Infectious Diseases, Rockefeller Branch, The Rockefeller University, New York, 10065 USA; 11 Pediatric Hematology-Immunology Unit, Necker Hospital for Sick Children, Paris, France; 12 Howard Hughes Medical Institute, New York, USA; 13 Rare Disease Center, Children’s Hospital, University of Helsinki and Helsinki University Hospital, Helsinki, Finland; 14 Adult Immunodeficiency Unit, Department of Infectious Diseases, Inflammation Center, University of Helsinki and Helsinki University Hospital, Helsinki, Finland

**Keywords:** Telomere biology disorders,Telomeropathies, Next-generation sequencing, Whole-exome sequencing, Dyskeratosis congenita, *DKC1*; *TERT*; *RTEL1*

## Abstract

**Background:**

The telomere biology disorders (TBDs) include a range of multisystem diseases characterized by mucocutaneous symptoms and bone marrow failure. In dyskeratosis congenita (DKC), the clinical features of TBDs stem from the depletion of crucial stem cell populations in highly proliferative tissues, resulting from abnormal telomerase function. Due to the wide spectrum of clinical presentations and lack of a conclusive laboratory test it may be challenging to reach a clinical diagnosis, especially if patients lack the pathognomonic clinical features of TBDs.

**Methods:**

Clinical sequencing was performed on a cohort of patients presenting with variable immune phenotypes lacking molecular diagnoses. Hypothesis-free whole-exome sequencing (WES) was selected in the absence of compelling diagnostic hints in patients with variable immunological and haematological conditions.

**Results:**

In four patients belonging to three families, we have detected five novel variants in known TBD-causing genes (*DKC1*, *TERT* and *RTEL1*). In addition to the molecular findings, they all presented shortened blood cell telomeres. These findings are consistent with the displayed TBD phenotypes, addressing towards the molecular diagnosis and subsequent clinical follow-up of the patients.

**Conclusions:**

Our results strongly support the utility of WES-based approaches for routine genetic diagnostics of TBD patients with heterogeneous or atypical clinical presentation who otherwise might remain undiagnosed.

**Electronic supplementary material:**

The online version of this article (10.1186/s13023-018-0864-9) contains supplementary material, which is available to authorized users.

## Background

The telomere biology disorders (TBDs), or telomeropathies, embody a range of pathological phenotypes ensuing from abnormal telomerase function. The first TBD to be described was dyskeratosis congenita (DKC), a severe inherited multisystem disorder characterized by reticulate skin pigmentation, nail dystrophy, oral leukoplakia and bone marrow failure, presenting with cytopenia of one or more hematopoietic cell lineages [1]. Clinically, telomere shortening due to the premature senescence of stem cells is most prominently displayed in highly proliferating mucocutaneous tissues [2]. The most severe clinical feature of DKC is bone marrow failure, affecting the majority of patients and causing premature mortality. In addition, DKC patients have an increased risk for malignancies, fatal pulmonary complications, and immunodeficiency [3]. At present, DKC is known to be caused by mutations in 11 genes, associated with X-linked recessive inheritance in *DKC1*, or with autosomal recessive and/or dominant inheritance in *TERT*, *TERC*, *NHP2*, *NOP10*, *ACD*, *WRAP53*, *TINF2*, *RTEL1*, *CTC1*, and *PARN* [2, 4–6]. Recently, the term DKC has been used to categorize only well-defined childhood symptoms [4]. Based on the phenotype, a severe variant of DKC manifests as Hoyeraal-Hreidarsson syndrome (HHS). This is a rare disorder characterized by bone marrow failure, immunodeficiency, cerebral hypoplasia and intra-uterine growth retardation [3, 7]. DKC has often gone unnoticed due to delayed-onset of mucocutaneous findings [8]. The wide spectrum of clinical presentations and the lack of a conclusive laboratory test for DKC can at times make the clinical diagnosis challenging. Exact genetic diagnosis in DKC is essential due to the limited efficacy of therapeutic options, and the genetic anticipation common in DKC makes timely family counseling a priority.

In the present study, we attested the validity of whole-exome sequencing (WES)-based approaches for routine genetic diagnostics of patients with heterogeneous clinical presentation or suspicion of TBD. In three patients and a brother of one of the patients presenting with varied characteristics of immunological or hematological disorders, we identified monogenic variants in genes associated with TBDs, and supported the genotype-phenotype correlation by demonstrating shortened blood cell telomere length.

## Methods

### Study subjects

This study was performed in Helsinki University Hospital, at the Pediatric and Adult Immunodeficiency Units, at the Department of Adult Hematology and at the Division of Pediatric Hematology-Oncology and Stem Cell Transplantation. Genetic analyses were performed as part of clinical workup in 212 clinically undiagnosed patients with disease phenotypes suggestive of monogenic conditions overlapping Primary immunodeficiency disorders (PIDs). The characterization of the phenotypes of the studied patients is shown in the Additional file 1: Figure S1. After the identification of alterations in TBD-associated genes in Finnish patients, we retrospectively collected their clinical data. This study was conducted in accordance with the principles of the Helsinki Declaration and was approved by the Coordinating Ethics Committee of Helsinki University Hospital. An informed consent was received from patients and in case of children, from their parents.

### Molecular genetics

Genomic DNA of the studied individuals was isolated from peripheral blood through standard salt precipitation protocols. Whole-exome sequencing (WES) was performed in 212 patients. The detailed workflow for reads alignment and variant calling is provided in the Additional file 2.

Briefly, a SureSelect Clinical Research Capture Exome or SureSelect Human All Exon 50 Mb kits (Agilent, Santa Clara, CA, USA) were used. Paired-end sequencing was performed on the HiSeq 1500 or HiSeq 2000 platforms (Illumina, San Diego, CA, USA). The sequencing reads were analyzed using version 2.7 of the in-house developed analysis pipeline (VCP) for quality control and variant identification [9]. Annovar (accessed in May 2017) was used for the annotations and prediction of functional consequences of the identified variants [10, 11]. The sequences were aligned with the GRCh39 reference build of the human genome using the BWA aligner [12]. Downstream processing and variant calling were performed with the Genome Analysis Toolkit [13], SAMtools [14], and Picard. Substitution and InDel calls were made with GATK Unified Genotyper.

We considered only variants with a minor allele frequency (MAF) < 0.01, with frequency filtering based on data from Genome Aggregation Database (gnomAD, Cambridge, MA, USA; http://gnomad.broadinstitute.org/; and the *SISu* project (http://sisu.fimm.fi/) accessed in May 2017, respectively [15, 16]. We filtered according to the predicted consequences at the transcript level, selecting frameshift, in-frame, nonsense, splicing and missense variants. The variants were prioritized according to the predicted effect on the protein, to the conservation of the affected amino acids and in silico prediction tools (included in Annovar), and pathogenicity was predicted according to the American College of Medical Genetics (ACMG) Standards and Guidelines [17]. Where possible, the candidate variants were analyzed in the families for co-segregation with affected relatives. Due to the several described inheritance models, we considered homozygous, compound heterozygous and heterozygous variants. All identified candidate variants were validated by Sanger sequencing (Additional file 2).

### Telomere length analysis

Relative telomere length (RTL) was determined by the quantitative PCR method described by Cawthon et al. [18], with minor modifications. Briefly, each sample (17.5 ng DNA from peripheral blood leukocytes) was analyzed in triplicate wells in separate telomere (TEL) and single copy gene (HBG) reactions on an ABI 7900HT instrument (Applied Biosystems), at two separate occasions in 96-well PCR plates. TEL/HBG (T/S) values were calculated by the 2 − ΔCt method, where ΔCt = Ct_TEL_-Ct_HBG_. The RTL value was generated by dividing samples T/S value with the T/S value of a reference cell-line DNA (CCRF-CEM) included in all runs. A standard curve of the reference cell line DNA was included in every run to monitor PCR efficiency. The RTL values were compared to 113 normal controls (age 0–83 years).

## Results

**Fig. 1:**
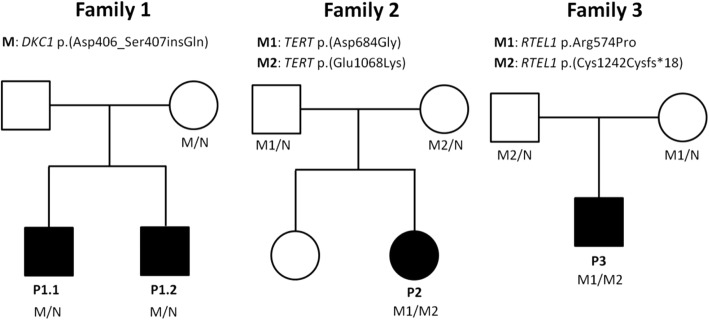
Pedigrees of three families with telomere biology disorders. The panel shows pedigrees of patients with telomere biology disorders (TBD). The original index cases are indicated as P1.1-P3. Solid symbols indicate affected patients, open symbols unaffected. For each family, the identified variants in TBD-associated genes are indicated by M. Normal alleles are listed as N

Genetic screening of 212 clinically undiagnosed Finnish patients identified five novel disease variants in TBD-associated genes in three patients and a brother of one of the patients (Fig. 1, Tables 1 and 2), most of which with no clear pathognomonic signs of telomeropathies. The hematological and immunological characterizations of the patients are summarized in Table 3.

**Table 1:** Demographic and clinical data of patients with germline variants in genes associated with telomere biology disorders

Patient	Sex	Age	Gene variant	Family history	Skin, nail anomalies	Hair graying/ loss	Oral mucosal changes	Aplastic anemia/ cytopenias	Immuno-deficiency	Pulmonary fibrosis	Infertility	Gastro-intestinal problems	Developmental defects	Relative telomere length^a^
P1.1	M	24	*DKC1*	*+*	+	–	+	–	+	–	NA	–	–	short
P1.2	M	28	*DKC1*	*+*	+	–	+	–	+	–	NA	–	–	short
P2	F	11	*TERT*	*–*	–	–	–	+	–	–	NA	+	+	short
P3	M	2	*RTEL1*	*–*	+	–	–	–	+	–	NA	+	+	short

Abbreviations: F, female; M, male; +, feature present; −, feature absent; NA, not available

^a^Compared to 113 healthy controls (age 0–83 years)

**Table 2:** Germline variants identified in patients with telomere biology disorders

ID	Gene	Nucleotide change^a^	Amino acid change^a^	Inheritance	MAF^b^	Carriers (overall / Finland)^b^	Affected domain^c, **^	REVEL score^d, **^	Classification^e^	Reference
P1.1	*DKC1*	c.1218_1219insCAG	p.(Asp406_Ser407insGln)	XLR^f^	–	–	–	–	Likely pathogenic	novel
P1.2	*DKC1*	c.1218_1219insCAG	p.(Asp406_Ser407insGln)	XLR^f^	–	–	–	–	Likely pathogenic	novel
P2	*TERT*	c.2051A > G	p.(Asp684Gly)	AR	0.0015	27/25	Reverse transcriptase domain	0.548	Uncertain significance	novel
	*TERT*	c.3202G > A	p.(Glu1068Lys)	AR	–	–	–	0.546	Uncertain significance	novel
P3	*RTEL1*	c.1721G > C	p.Arg574Pro	AR	4.08E-06	1/0	ATP-dependent helicase, C-terminal; P-loop containing nucleoside triphosphate hydrolase	0.752	Likely pathogenic	novel
	*RTEL1*	c.3724_3725delTG	p.(Cys1242Cysfs^f^18)	AR	8.20E-06	2/1	–	–	Pathogenic	novel

Abbreviations: F, female; M, male; XLR, X-linked recessive, autosomal recessive; MAF, minor allele frequency

**data retrieved with Annovar; AR

*DKC1* gene reference sequences (Ensembl): ENSG00000130826; ENST00000369550

*TERT* gene reference sequences (Ensembl): ENSG00000164362; ENST00000296820

*RTEL1* gene reference sequences (Ensembl): ENSG00000258366; ENST00000318100

^a^location according to RefSeq

^b^minor allele frequency according to gnomAD database**

^c^InterPro database**

^d^REVEL pathogenicity score** [26]

^e^estimated according to the ACMG Standards and Guidelines [17]

^f^male

**Table 3:** Summary of immunologic features of the patients with telomere biology disorders

Patient	Reference Range (adults/children)	P1.1	P1.2	P2	P3
Lymphocytes	1300–3600/1700- 6900/1100–5900×106/L	2030	2340	660	1370
Monocytes	200-800 × 10^6^/L	620	560	290	460
Neutrophils	1500-6700 × 10^6^/L	1750	2550	1110	6540
Basophils	0-100 × 10^6^/L	10	40	10	20
Eosinophils	30-440 × 10^6^/L	90	170	7	40
Platelets	150,000–360 000 × 10^6^/L	218,000	264,000	62,000	233,000
B-cells (CD19+)	200–2100/ 200–1600×10^6^/L	200	360	80	50
CD3+	900–4500/700–4200× 10^6^/L	1340	1670	560	490
CD3 + CD4+	500–2400/ 300–2000×10^6^/L	430	817	303	294
CD3 + CD8+	300–1600/00–1800×10^6^/L	770	800	230	220
NK-cells (CD3^−^CD16^+^ 56^+^)	100–1000/ 90–900×10^6^/L	140	200	90	20
Plasmacytoid	0.1–0.3%	0.14	NA	0.10%	NA
(lin^−^HLA-DR^+^CD123^+^CD11c^−^)					
Monocytoid	0.1–0.3%	0.42	NA	0.24%	NA
(lin^−^HLA-DR^+^CD123^−^CD11c^+^)					
IgG	6.8–15.0 g/L	11.1	NA	14.3	1.9
IgA	0.52–4.02 g/L	2.95	NA	2.82	0.39
IgM	0.47–2.84 g/L	0.47	NA	1.44	0.5
IgE	0–110 IU/L	46	NA	NA	< 2
Lymphocyte proliferative responses to mitogens	Phytohemagglutinin Concanavalin A	normal	NA	normal	abnormal

Abbreviations: NA, not assessed

Patient 1 (P1.1) is a 24-year-old male from a non-consanguineous family. He was remitted to WES to search for mutations associated with chronic mucocutaneous candidiasis, with the identification of a novel variant in *DKC1* (c.1218_1219insCAG, p.(Asp406_Ser407insGln)). In early childhood, he had been diagnosed with vesicoureteral reflux and urinary tract infections, but never suffered from severe infections. He received normal vaccinations including MMR. From an early age on, his fingernails were abnormal and broke easily. His skin was reddish and easily irritable. The skin changes in his neck and upper thorax were consistent with poikiloderma. At the age of 10–13 years, he started presenting with a recurrent aphthous ulcer on oral mucosa including the tongue. Patchy lesions of homogenous leukoplakia were observed on the dorsum of the tongue and the ventral side. In biopsies, epithelial changes compatible with leukoplakia and candidiasis were found resulting in the immunological workup. He had slightly decreased IgG2 concentration and CD4+ T cell count but otherwise normal results. He had no other cytopenias. At the age of 19 years, Th17 cells were found to be subnormal (< 0.02% of CD4 T cells), but this could not be confirmed in retesting. Family studies showed that an older brother (P1.2) of the index patient suffered from slightly milder skin, nail and oral mucous membrane abnormalities, as well as recurrent genital *Candida* infections. Genetic testing revealed that P1.2 carried the same variant, confirming co-segregation with the disease. The variant was inherited from the mother, consistent with X-linked recessive inheritance. The maternal grandfather died traumatically at his 40’s but is remembered to have had nails and skin changes, as well. To the best of our knowledge, none of the close family of P1.1 and P1.2 has suffered from solid tumors, hematological malignancy, lung fibrosis or peripheral blood cytopenias.

Patient 2 (P2) is an 11-year-old daughter of non-consanguineous parents. The WES analysis revealed two novel heterozygous variants in *TERT*: c.2051A > G, p.(Asp684Gly) and c.3202G > A, p.(Glu1068Lys). Segregation analysis in the family demonstrated that the patient had inherited one variant from each parent, supporting autosomal recessive inheritance. She was born at term but small-for-gestational-age and catching up the normal growth by the age of two. No developmental delays were recorded early in the infancy or later on. At the age of four, she had disorders in swallowing, and oesophageal strictures were diagnosed requiring dilatation. By the same age, mild thrombocytopenia and macrocytosis were detected in the peripheral blood, but the bone marrow showed normal hematopoiesis. As the cytopenias persisted, re-evaluation of her bone marrow at the age of 10 years showed features of myelodysplasia and hypoplastic anemia. Paroxysmal nocturnal hemoglobinuria and Fanconi anemia were excluded. Despite extensive immunological evaluation, no immunodeficiency has been detected. She has never presented pulmonary, nail or skin symptoms. She has fair and thin hair. Her older sister (16-years old) did not present any symptoms. None of her close relatives were diagnosed with cancer or classical DKC.

Patient 3 (P3) is a 2-year-old boy of non-consanguineous parents. He was analyzed by WES due to immunodeficiency and the occurrence of opportunistic infections, identifying two novel heterozygous variants in *RTEL1*: c.1721G > C, p.Arg574Pro (confirmed at RNA transcript level) and c.3724_3725delTG, p.(Cys1242Cysfs*18). Both variants are rare in the population and segregation analysis showed that one variant was inherited from each parent, supporting autosomal recessive inheritance. He was born prematurely at gestational week 31 and was small-for-gestational-age (weight − 3.6 SD/ height − 2.2 SD). He received normal vaccinations at 3 months of age. At the age of 7 months, he was hospitalized for fever and pneumonia, and *Pneumocystis jirovecii* plus metapneumovirus and coronavirus were detected in the respiratory secretions. In addition, cytomegalovirus DNAemia was present with high copy numbers accompanied by retinitis. Immunological work-up revealed a T^−^B^−^NK^−^ severe combined immunodeficiency (SCID) phenotype. Brain MRI showed atrophic changes in the cerebellum and microcephalia. At the age of 9 months, hematopoietic stem cell transplantation (HSCT) with umbilical cord graft was performed due to SCID. Engraftment was with full donor chimerism without signs of graft-versus-host disease. Transient CMV reactivation was successfully treated with antiviral treatments. He had prolonged diarrhea preceding the HSCT, and colitis-like symptoms continued after HSCT needing long-lasting total parenteral nutrition. Otherwise, with the recovering immunity, his pulmonary problems abated and there were no signs of decreased liver function. So far, due to young age and HSCT performed with chemotherapeutic conditioning, we cannot reliably evaluate the status of the hair and skin of this patient. After HSCT, he has been treated by a pediatric neurologist and the parents have received genetic counseling. There is no known family history of immunodeficiency or malignancy.

### Telomere length analysis

**Fig. 2:**
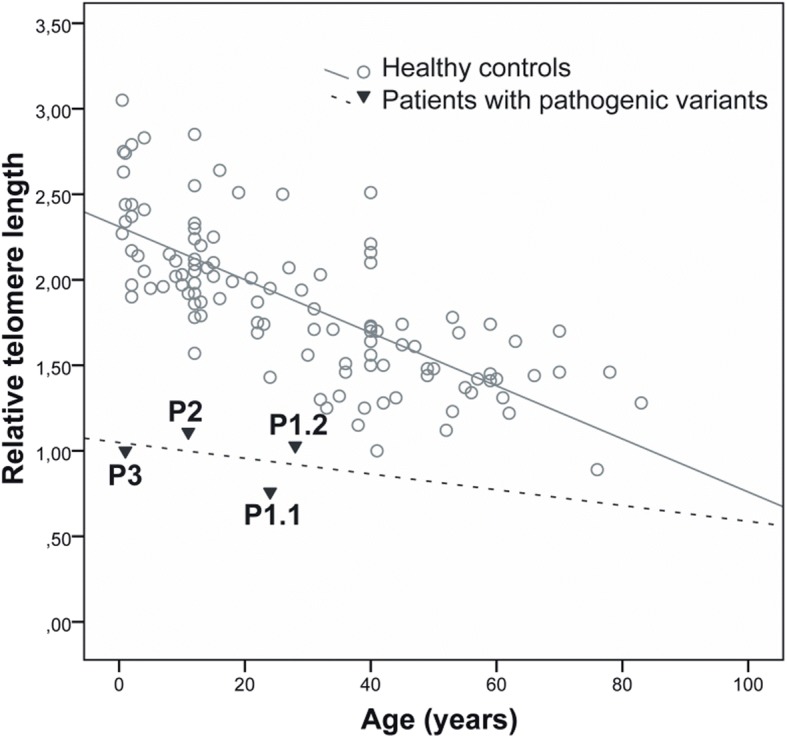
Relative telomere length of the patients with telomere biology disorders. The relative telomere length (RTL) value of each sample (Y-axis) is plotted against the individual’s age (X-axis). Solid triangles indicate the four TBD-patients of the study (P1.1-P3). Open circles represent the 113 healthy controls (age 0–83 years) used for the comparison.

Due to the identification of variants in genes associated with defective telomere function, we analyzed the length of the telomeres in the four patients. All patients (P1.1, P1.2, P2, and P3) showed short relative telomere length below the 5th percentile compared to healthy controls of the same age (Fig. 2). The integration of genetic findings, clinical phenotype, and decreased telomere length led to a diagnosis of DKC in P1.1 and P1.2, bone marrow failure in P2, and Hoyeraal-Hreidarsson syndrome in P3.

## Discussion

In the present study, by using hypothesis-free WES we identified disease variants in telomeropathy-associated genes in four patients with clinical phenotypes ranging from mild signs of DKC to SCID.

Telomeropathies include a range of pathological conditions resulting from defective telomere biology, with great variation in terms of clinical severity and age at diagnosis [19]. Not all TBD patients display the classical triad of symptoms (skin pigmentation, nail dystrophy and oral leukoplakia), thus lacking the pathognomonic signs of DKC. In some patients, bone marrow failure or immunodeficiency may be the only presenting symptom. The most severe phenotypical variant of DKC, Hoyeraal-Hreidarsson syndrome (HHS), may clinically present as SCID in children [7]. However, bi-allelic mutations in the HHS-causing gene *RTEL1* may also lead to selective NK deficiency [20]. Wide phenotypic variation may contribute to the diagnostic delay, especially in adult patients, as seen in P1.1 and P1.2 who were not diagnosed until the age of 24 and 28 years. In addition to the diverse clinical presentation, mutations in various genes causing telomere-shortening may manifest with increasing severity and shorter telomeres in successive generations, a process called genetic anticipation [21]. Thus, TBD patients with mild phenotype may have offspring potentially manifesting early in life with bone marrow failure or severe immunological aberrations with aggravated complications like rapid deterioration of pulmonary function. Due to the increasing severity in further generations, these disease-causing variants tend to eventually disappear from the pedigree. Likely for this reason, most of the Finnish families with telomeropathies included in this study appeared to have rare, novel mutations. This is fully opposite to the majority of the rare recessive diseases seen in Finland, which are typically caused by one major Finnish founder mutation enriched in the population [22, 23]. Therefore, the possibility of a novel variant needs to be considered for patients with TBD suspicion where no known pathological variants have been found in a targeted-variants approach.

Due to the heterogeneous clinical presentations and the allelic heterogeneity, patients with clinical suspicion of telomeropathies could benefit from the investigation by WES or other applicable NGS-based assays. This is successfully demonstrated in this study, where only one patient within our cohort (P1.1) presented with the classical skin, nail and mucosal findings suggestive of DKC. Furthermore, for one patient (P3) the genetic analysis immediately produced the final molecular diagnosis of HHS, while the initially observed clinical features (SCID) could have led to the use of repeated mistargeted assays not identifying the causal variants.

The identification of variants in TBD-associated genes in patients showing seemingly distinct immune or haematological conditions emphasizes the potential of WES for discerning atypical traits of monogenic syndromes and broadening the phenotypic spectrum of the disease in addition to providing a prompt molecular diagnosis. WES also allows screening for secondary causative variants potentially explaining the untypical phenotypic features. Importantly, WES combined with telomere length measurement further enables identification of novel TBD causing gene defects, given that novel TBD-causing genes are yet to be discovered. Because of the genetic heterogeneity, measurement of telomere length can also be considered for screening of undiagnosed patients with clinical features compatible with TBDs. The quantitative PCR-based assay used in this study is a cost-efficient mean to complement the diagnostics in patients with bone marrow failure or primary immunodeficiency.

From the genetic point of view, early diagnosis of TBD is critical for the counseling provided to the families. Once the disease-causing mutation(s) have been identified, assessment of allelic segregation should be systematically recommended to the entire family, particularly in case of X-linked TBD. As exemplified by *DKC1*, the disease may be carried silently in females and transmitted to next generations in absence of prenatal diagnosis. Also, some women may present with symptoms due to skewed X-inactivation, therefore further studies of the families are indicated [24]. From the clinical point of view, the treating hematologists should consider the possibility of TBDs when evaluating patients with bone marrow failure, apparently unexplained cytopenias or myelodysplastic syndrome emerging at an early age, even in the absence of the classic mucocutaneous triad. Prompt diagnosis of a telomeropathy in case of atypically presenting TBD, like aplastic anemia, is important to bring to attention the need of monitoring other possibly arising TBD-related organ complications. In addition, immunosuppressive therapy is unlikely to be beneficial, but patients may profit from treatment with anabolic steroids aiming at improving cell counts. Furthermore, patients with severe bone marrow failure can be evaluated for hematopoietic stem cell transplantation. Knowledge of exact genetic diagnosis will help in planning the preparative regimen and follow-up after HSCT, as well as in the choosing of donors among family members.

## Conclusions

Using hypothesis-free WES we identified novel disease variants in TBD-associated genes in clinically undiagnosed Finnish patients. The use of WES helped the clinicians to provide a prompt diagnosis of telomeropathy in the absence of the classic pathognomonic signs. We highlight the relevance of achieving a molecular diagnosis and subsequent accurate genetic counseling since effective treatments are lacking for TBDs.

## Additional files

Additional file 1: **Figure S1.** Categorization of the clinical phenotypes of the patients in the studied dataset (*n* = 212 patients). Categories defined according to the 2017 Primary Immunodeficiency Disease Committee Report [25]. (JPG 14 kb)

Additional file 2: Supplementary methods the detailed description of the methods used for the molecular genetics analyses included in this paper. (DOC 41 kb)

## Abbreviations

*ACD*: ACD, Shelterin Complex Subunit And Telomerase Recruitment Factor
CD4: Cluster of differentiation 4
CMV: Cytomegalovirus
*CTC1*: CST Telomere Replication Complex Component 1
DKC: Dyskeratosis congenita
*DKC1*: Dyskerin Pseudouridine Synthase 1
HHS: Hoyeraal-Hreidarsson syndrome
HSCT: Hematopoietic stem cell transplantation
IgG2: Immunoglobulin G class 2
InDELS: Insertions or deletions
MAF: Minor allele frequency
Mb: Megabases
MMR: Measles, mumps, and rubella vaccine
ng: Nanograms
*NHP2*: NHP2 Ribonucleoprotein
NK: Natural killer cells
*NOP10*: NOP10 Ribonucleoprotein
*PARN*: Poly (A)-Specific Ribonuclease
PCR: Polymerase chain reaction
PIDs: Primary immunodeficiency disorders
*RTEL1*: Regulator Of Telomere Elongation Helicase 1
RTL: Relative telomere length
SCID: Severe combined immunodeficiency
SD: Standard deviation
*TERC*: Telomerase RNA Component
*TERT*: Telomerase Reverse Transcriptase
Th17: T helper 17
*TINF2*: TERF1 Interacting Nuclear Factor 2
WES: Whole-exome sequencing
*WRAP53*: WD Repeat-Containing Antisense To TP53
