# Supplementary figures and images for: Diagnostics of rare disorders: whole-exome sequencing deciphering locus heterogeneity in telomere biology disorders
Source: Orphanet J Rare Dis. 2018 Aug 17;13:139. doi: 10.1186/s13023-018-0864-9 (PMC6097299; doi:10.1186/s13023-018-0864-9)

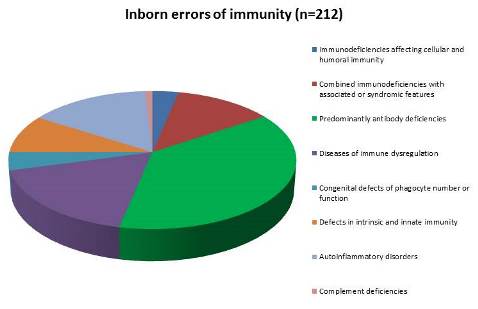

Supplement: Supplementary file 1 — Figure S1. Categorization of the clinical phenotypes of the patients in the studied dataset (n = 212 patients). Categories defined according to the 2017 Primary Immunodeficiency Disease Committee Report [25]. (JPG 14 kb) [file 13023_2018_864_MOESM1_ESM.jpg]
