# Supplementary material for: Diagnostics of rare disorders: whole-exome sequencing deciphering locus heterogeneity in telomere biology disorders
Source: Orphanet J Rare Dis. 2018 Aug 17;13:139. doi: 10.1186/s13023-018-0864-9 (PMC6097299; doi:10.1186/s13023-018-0864-9)
Supplement: Supplementary file 2 — Supplementary methods the detailed description of the methods used for the molecular genetics analyses included in this paper. (DOC 41 kb) [file 13023_2018_864_MOESM2_ESM.doc]

**Supplementary methods to:**

**Diagnostics of rare disorders: whole-exome sequencing deciphering locus heterogeneity in telomere biology disorders**

Luca Trotta, Anna Norberg, Ulla Wartiovaara-Kautto, Vivien Béziat, Sofie Degerman, Mervi Taskinen, Hannamari Välimaa, Kirsi Jahnukainen, Jean-Laurent Casanova, Mikko Seppänen, Janna Saarela, Minna Koskenvuo, Timi Martelius

**Molecular genetics**

DNA and RNA for genetic analyses were extracted from peripheral blood using standard methods. Whole-exome sequencing (WES) was executed in 212 patients using the SureSelect Human All Exon 50 Mb kits (Agilent, Santa Clara, CA, USA) for the capture. Paired-end sequencing was performed on the HiSeq 200 (P1.1) or HiSeq 1500 (P2, P3) platforms (Illumina, San Diego, CA, USA). The sequences were aligned with the GRCh39 reference build of the human genome using the BWA aligner [14]. Downstream processing and variant calling were performed with the Genome Analysis Toolkit [15], SAMtools [16], and Picard. Substitution and InDel calls were made with GATK Unified Genotyper. The WES data were analyzed using a version 2.7 of the in-house developed analysis pipeline for quality control and variant identification (VCP). [11] Raw Illumina reads were first merged with SeqPrep (0.4.5). The resulting paired reads were trimmed of # blocks in the quality scores from the end of the read. After this, any read shorter than 36 base pairs was removed. The paired reads and single reads were aligned separately using the Burrows Wheeler Aligner (version 0.5.10) against the human genome (ensembl version 70). The alignment was refined using GATK Indel Realignment (version 1.5-3). After the alignment, potential PCR duplicates were removed with Picard MarkDuplicates (version 1.65). Also, any read pair where both reads were mapping or any single reads which was mapping to multiple genomic positions were removed. Variant calling was performed with SAMtools mpileup (version 0.1.18) and dindel (version 1.01). The SNPs were called with minimum depth of 7. The resulting SNPs were then recalculated using quality values. A ratio between sum of quality values of reference (R) and variant (V) calls was calculated: R / (R + V), discarding any where the ratio was above 0.8. If there were two variant calls and no reference, the call with higher quality value was used in place of reference. Any call with ratio smaller than 0.2 was assumed to be homozygous and the rest heterozygous. Annovar was used for the annotations and prediction of functional consequences of the identified variants. [12, 13]

All the common (frequencies above 0.01 in the general population) and non-coding variants were discarded. The frequency filtering were based on data from Genome Aggregation Database (gnomAD, Cambridge, MA, USA; http://gnomad.broadinstitute.org/; accessed in May 2017) and the *SISu* project (http://sisu.fimm.fi/). [14, 15] The rare variants affecting the coding regions were filtered based on the predicted consequences at the transcript level, with the selection of frameshift, nonsense, splicing and missense variants, then analyzed in the familial frame using in-house designed IT procedures. Due to the potential autosomal-recessive and dominant inheritance models, we retrieved the homozygous, compound heterozygous and heterozygous variants shared by the patients (Supplementary Figure 1A). The variants were evaluated according to the ACMG Standards and Guidelines [17] and prioritized using the predicted effect on the protein, the conservation of the affected aminoacids and *in silico* prediction tools. In particular, the pathogenic effect of the detected variants was estimated through the REVEL score, ranging from 0 (non pathogenic) to 1 (potential pathogenic effect). [24] In addition, we ruled out variants in the reported TBD-causing gene *TERC* [4]. *TERC* does not encode for a protein, therefore any potential variants in the gene are filtered out based on the predicted consequences at the transcript level. The ACMG Recommendation for reporting of incidental findings were applied to WES data of P1.1, P2 and P3, without any emerging variant [17].

All the identified candidate variants were confirmed by Sanger sequencing of genomic DNA. In addition, the *RTEL1* variants identified in P3 were investigated at the transcript level by Sanger sequencing of cDNA. The cDNA was obtained from RNA using the SuperScript® VILO cDNA Synthesis kit (Life Technologies, Carlsbad, CA) according to the manufacturer’s instructions (using 0.5-2µg of RNA in a 20µl reaction volume). Sanger sequencing was performed using the ABI-3730XL DNA Analyzer and BigDye Terminator Cycle Sequencing kit (Applied Biosystem, Foster City, CA, USA). The sequences of forward and reverse primers used for the validation of the final set of variants are available upon request. The Sanger sequencing data was analyzed using Sequencer 5.1 (Gene Codes Corporation, Ann Arbor, MI USA).
